# Effectiveness of a Haemorrhage-Control Task Simulator for Training Nursing Students: A Quasi-Experimental before-after Study

**DOI:** 10.1155/2024/9730765

**Published:** 2024-03-13

**Authors:** Daniel Bertini-Pérez, Luis Martin-Ibañez, Pablo Gómez Chica, Iria Dobarrio-Sanz, Miguel Rodriguez-Arrastia, Pablo Roman, Lola Rueda-Ruzafa

**Affiliations:** ^1^Faculty of Health Sciences, Department of Nursing, Physiotherapy and Medicine, University of Almeria, Almeria 04120, Spain; ^2^Field Artillery Group, Light Infantry Brigade “King Alfonso XIII” II of the Legion, Almeria 04240, Spain; ^3^Research Group CTS-451 Health Advances and Innovation, University of Almeria, Almeria 04120, Spain; ^4^Health Sciences Advanced and Innovation Research Group (CTS-1114), Almeria 04120, Spain; ^5^Science Flows, Universitat de València, Valencia 46010, Spain

## Abstract

**Aim:**

To assess the efficacy of a low-cost haemorrhage-control task simulator integrated in a high-fidelity simulation scenario to facilitate knowledge and practical skills acquisition, as well as self-efficacy in haemorrhage control among nursing students.

**Design:**

A quasiexperimental before-after design was conducted at the University of Almeria.

**Methods:**

A one-group preintervention, immediate postintervention, and a third assessment at three months were performed, with the Stop the Bleed Education Assessment Tool used to evaluate knowledge of haemorrhage control, as well as a 5-point Likert scale used to evaluate perceived self-efficacy. The success of controlling exsanguinating bleeding was determined by quantifying the millilitres lost during the intervention and calculating the time required to control the haemorrhaging. The data were reported using the TREND guidelines.

**Results:**

One hundred and three final-year nursing students participated in this study. Significant improvements (*p* < 0.001) were observed in pre- and posttest total scores on knowledge of bleeding control, self-efficacy, as well as time is taken and volume loss to control the haemorrhage. Similar results were observed between preassessment and three months postassessment with significant improvements (*p* < 0.001) in all measures.

**Conclusions:**

The use of a haemorrhage-control task simulator within a high-fidelity simulation scenario resulted in noteworthy improvements in nursing students' practical skills, knowledge retention, and self-efficacy. After three months, performance decreased but remained greater than pretraining levels. Thus, broadening the use of this task-training simulator would be of great value to further develop a first responder training approach with healthcare professionals and other laypersons, allowing for greater knowledge distribution and reaching a larger audience. *Implications for Nursing Management*. The findings underscore the potential efficacy of this simulator as a valuable resource for nursing educators and supervisors to train nursing students and professionals in terms of practical skills, knowledge retention, and self-efficacy in haemorrhage control, fostering a train-the-trainer cascade approach to reach a wider audience and enhance bleeding control proficiency among professionals.

## 1. Introduction

Haemorrhagic shock is a severe form of hypovolemic shock that occurs when significant blood loss leads to insufficient oxygen delivery at the cellular level and, if ignored, may culminate in death within minutes [1]. Indeed, only haemorrhage is the cause of death in 55.1% of patients aged 1 to 46 [2], thereby being the primary cause of intrahospital mortality within 48 hours after admission and the second highest cause of prehospital death in both military and civilian trauma patients, accounting for 40–45% of all fatalities [3–6]. In this context, managing patients with haemorrhagic shock remains difficult and complex, with a high mortality rate [7–9]; hence, early recognition and prompt action to halt bleeding are critical, as the condition is time-dependent [4, 10].

Moreover, 34.5% of traumatic accident patients die from preventable bleeding-related causes, either in the prehospital setting or within an hour of being admitted to the hospital [11, 12]. While strengthening hospital and prehospital care seems to be essential for reducing trauma-related mortality, further bleeding control training needs to be provided to ensure such successful interventions [13–15]. The World Health Organisation and numerous consensus groups recommend first responder training programmes, for instance, as the first step in formalising emergency medical services in areas where no prehospital services have yet been created [16–18]. In this vein, one of the most effective and well-established training programmes for adequate bleeding control worldwide is the Stop the Bleed (STB) campaign, a basic short educational programme for both laypeople and healthcare professionals, that aims to provide education on wound compression, wound packing, and the use of emergency tourniquets [19]. According to certain research, even a single 2-hour STB training course could significantly increase self-confidence and competence among healthcare students instructed in bleeding control measures [20, 21].

Aside from the STB programme, there are a number of additional training programmes available that have similar objectives, which include Haemorrhage Control Course [22], Tactical Combat Casualty Care, and Advanced Trauma Life Support [23]. These programmes all share an agreed-upon aim of providing individuals with the knowledge and skills to effectively manage haemorrhage, with tourniquets standing as the primary device used to control time in exsanguinating haemorrhage in extremities [24, 25]. Although different methods can be employed for this training, simulation-based education, ranging from low-fidelity simulation (LFS) to high-fidelity simulation (HFS), has been shown as one of the most successful teaching-learning approaches for these scenarios [26–28]. In this manner, high-fidelity simulation (HFS) stands as the most popular and effective approach for incorporating realism and authenticity into the educational experience for demanding scenarios [29]. Notably, nursing students demonstrate superior outcomes in both technical and nontechnical skills when employing HFS during their emergency environment training [30]. This may not only foster self-confidence but also help in lowering anxiety levels among students [29, 31]. However, recent evidence suggests that its use in haemorrhage control training programmes such as STB appears to be currently limited and costly due to the nature of the specialist simulators and personnel skillset required [32].

Notwithstanding self-efficacy in bleeding control is paramount in successfully performing the intervention, and specialised training for first responders and healthcare professionals has shown improvement in confidence across all domains, thereby increasing the ability to manage severe active bleeding and pack a bleeding wound, and there is still scant evidence regarding the use of cost-effective HFS for haemorrhage education. For these reasons, the aim of this study was to assess the efficacy of a low-cost haemorrhage-control task simulator, recently patented by our research team (ES-1294309_U [33]) and integrated into an HFS scenario to facilitate knowledge and practical skills acquisition, as well as self-efficacy in haemorrhage control among nursing students.

## 2. Material and Methods

### 2.1. Design and Participants

A quasiexperimental one-group before-after design was conducted at the University of Almeria from February to July 2022, following the recommendations of the Transparent Reporting of Evaluations with Nonrandomized Designs statement (TREND) guidelines [34]. The study participants were selected through purposive sampling from final-year nursing students who met the following inclusion criteria: (1) were enrolled in clinical placement 6 and 7 modules, (2) attended the bleeding control training, and (3) signed the required informed consent forms prior to participation.

### 2.2. Clinical Simulation

An HFS scenario was designed using a low-cost haemorrhage-control task simulator developed by our research team (ES-1294309_U) and based on the INACSL of Best Practise Standards: SM Simulation Design framework [35]. This simulator comprises a first container, means for pressurizing the infusing liquid, a venepuncture member with a flexible conduit leading to a second collecting container, and pressure control means for the user to regulate the pressure on the venepuncture member. The simulator is calibrated to measure the millilitres exsanguinated and includes a timer to record the time taken to control the haemorrhage. Specifically, the device replicates a haemorrhage in a limb within a closed circuit, using a fluid simulating blood, pressurized at a constant pressure chosen by the user-trainer, optionally through a manual or automatic sphygmomanometer. Proper and effective tourniquet placement is essential for haemorrhage control, requiring the application of the appropriate pressure.

The clinical setting was intended to simulate an exsanguinating haemorrhage scenario in which the participant was only able to control the condition using a tourniquet, without prior training (PRE). The exsanguinating haemorrhage was caused by a 6-millimetre incised wound on the anterior surface of the simulator's forearm.  Table 1 summarises the required elements used to lead the simulation-based experience.

Following the collection of PRE data, the clinical simulation started with an initial 90-minute briefing in which participants were presented with the purpose of the intervention and the scenario they were facing. The nursing students received training on the proper management of exsanguinating haemorrhages using a tourniquet. This training was provided by experienced military nurses in the field and was based on the first-care provider model. After the initial briefing, the simulation stage began, in which the students attempted to control the haemorrhage with the tourniquet (POST), applying the training received during the clinical simulation by the military. This was followed by a 30-minute debriefing stage in which critical thinking and perceptions were shared collectively. Additionally, three months (3M) later, a third assessment of each study parameter was performed to assess the short- to medium-term retention of knowledge and practical skills for bleeding control.

### 2.3. Instruments

The sociodemographic data were collected using an ad hoc questionnaire created expressly for this purpose. The gender, age, and total amount of previous training in bleeding control were all questions on this questionnaire. The level of knowledge in bleeding control, success in haemorrhage control with tourniquet application, and students' training self-efficacy were measured as outcome variables pre- and postintervention.

Pre- and postintervention student knowledge was assessed using a nonvalidated modified version of the Stop the Bleed Education Assessment Tool (SBEAT) [36], a standardised assessment instrument for evaluating cognitive components of first aid for potentially life-threatening haemorrhages. The tool used incorporates 34 items into 20 survey questions. The questionnaire score ranged from 0 to 100, with a higher score indicating more knowledge in the field. In a Rasch model, the mean infit statistics were 1.00 and the outfit was 0.99, showing a reasonable level of fit [37].

Conversely, student self-efficacy was measured using a 5-point Likert scale, which was applied to 23 questions in the developed questionnaire. The responses to each of the questions were totalled up at the end of the questionnaire and shifted so that the maximum score was 100 points; a higher score on the questionnaire was interpreted as a greater perceived self-efficacy by the student. Cronbach's alpha reliability levels for this instrument were 0.99 (PRE), 0.99 (POST), and 0.98 (3M).

Lastly, the success of controlling exsanguinating bleeding was determined by quantifying the millilitres lost during the intervention and calculating the time required to control the haemorrhaging. The blood lost quantification was carried out by instructors directly observing the simulator's collection bag, which was graduated in 20-millilitre increments. When the amount of exsanguinated blood did not exceed 800 millilitres and the time did not exceed 120 seconds, the intervention was deemed successful.

### 2.4. Data Analysis

All statistical analyses of the data were conducted using the IBM SPSS Statistics v.27.0 software. A significant value of *p* < 0.05 was considered for all statistical tests performed. First, a descriptive analysis was performed on sociodemographic variables. The frequencies and percentages of qualitative variables, and the mean and standard deviation of quantitative variables were calculated. The Kolmogorov–Smirnov test was used to determine the normal distribution of the data. The chi-square test was then used to analyse the differences between qualitative variables. The Student's *t*-test or Mann–Whitney *U* test, ANOVA, or Kruskal–Wallis test were used for quantitative variables, depending on whether the variables matched normality conditions or not. Depending on the distribution, Pearson's or Spearman's correlation was used to relate quantitative variables.

### 2.5. Ethical Considerations

The study proposal was approved and authorised by the Ethics Committee of the Department of Nursing, Physiotherapy andMedicine at the University of Almeria (EFM 99/2021) and in accordance with all of the ethical aspects of the Declaration of Helsinki. All participants were informed and signed informed consent forms prior to participating in the study, as well as had the option to withdraw at any time. Added to that, participants were advised that their participation in the clinical simulation training would have no influence on their grades.

## 3. Results

One hundred and three final-year nursing students participated in the study, with women accounting for 78.64% (*n* = 81) and men for 21.36% (*n* = 22). In terms of age, the mean age was 23.34 years (5.81), with a range of 21 to 56 years. Baseline characteristics are summarised in Table 2.

**Figure 1:**
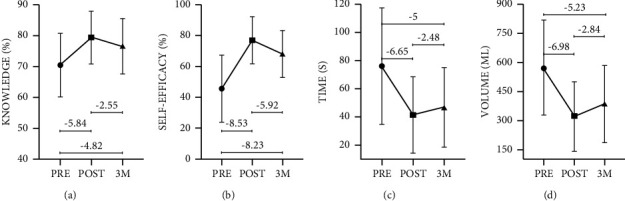
Comparison of the variables knowledge, self-efficacy, time, and volume pre- and postintervention.

Table 3 and Figure 1 provide a comparison of the variables knowledge, self-efficacy, time, and volume pre- and postintervention. These phases correspond to the initial evaluation (PRE), the immediate evaluation after the intervention (POST), and the three-month (3M) late evaluation.

In terms of participant knowledge, statistically significant differences were found between the PRE and POST findings (*Z* = −5.84; *p* < 0.01), as well as at 3M when compared to the PRE phase (*Z* = −4.82; *p* < 0.01). The mean questionnaire score in the PRE phase was 70.58 (10.24), increasing to 79.51 (8.53) in the POST phase and 76.70 (8.95) at 3M (Figure 1(a)).

PRE-intervention self-efficacy was significantly different from POST self-efficacy (*Z* = −8.53; *p* < 0.01) and self-efficacy findings at 3M (*Z* = −8.23; *p* < 0.01). The mean score in the PRE phase was 45.83 (21.69), 77.50 (15.24) in the POST phase, and 68.50 (15.19) at 3M (Figure 1(b)).

The time required for controlling bleeding differed statistically between the three phases: PRE-POST (*Z* = −6.65; *p* < 0.01), PRE-3M (*Z* = −5; *p* < 0.01), and POST-3M (*Z* = −2.48; *p* < 0.05). Prior to training, the mean time (s) to stop simulated haemorrhaging was 76.38 (41.28), which was reduced to an average of 41.69 (27.13) following the intervention and 47.13 (28.88) after three months (Figure 1(c)).

The difference in the amount of exsanguination (mL) in the simulator between the PRE and POST phases was statistically significant (*Z* = −6.98; *p* < 0.01) as was the difference between the PRE and 3M (*Z* = −5.23; *p* < 0.01) and between the POST and 3M (*Z* = −2.84; *p* < 0.01). The mean loss was 573.30 (246.01) in the PRE phase, 325.15 (179.83) in the POST phase, and 387.38 (199.10) at 3M (Figure 1(d)).

## 4. Discussion

The aim of our study was to assess the efficacy of a low-cost haemorrhage-control task simulator integrated into an HFS scenario to facilitate knowledge and practical skills acquisition, as well as self-efficacy in haemorrhage control among nursing students. While there have been a few studies in this field to date, the current study emphasises training utilising a low-cost simulator to improve clinical skills and knowledge retention in the nursing student training process, which is created from generally available materials, requiring a minimal economic investment.

According to the findings of our study, final-year nursing students had a very poor success rate in haemorrhage management. At baseline, only half of the study participants demonstrated effective control prior to the intervention using a tourniquet. Likewise, the time required and the number of millilitres of blood lost during the preintervention phase were inadequate. This supports previous findings employing certified training manikins as simulators [38, 39]. They found that the success rate in haemorrhage control among healthcare students was less than 66% and less than 24%, respectively, and the mean time taken was more than 75 seconds, and the amount of blood lost was more than 500 mL. Following the educational intervention, however, significant improvements in the level of knowledge, self-efficacy, and bleeding control skills among nursing students were observed, suggesting that the intervention was effective in improving the students' abilities to manage haemorrhages using a tourniquet. The present findings seem to be consistent with other research. Goralnick et al. [40] found that laypersons can successfully apply tourniquets following a one-hour course. Their study utilised various training methods, including an audio kit with visual aids on the device (audio kit) and instructional flashcards. Similarly, Stadeli et al. [41] reported improvements in knowledge and self-efficacy after participants received theoretical exposure with English slides followed by practical interpretation without an apparent simulator. Furthermore, Muret-Wagstaff et al. [42] observed that participants achieved a proficiency level in controlling bleeding after four sessions. Their training consisted of in-person instruction following the Peyton 4-stage model and simulation-based mastery learning with deliberate practice on certified simulators of amputated limbs [42]. While earlier research has noted the importance of educational interventions in the domain of self-efficacy in bleeding control training [43, 44], our findings have important implications for developing similar results in self-confidence and self-efficacy among nursing students utilising a low-cost simulator. In this vein and as mentioned in the literature, participants are able to attain nearly flawless competency scores and appropriately assist a bleeding victim by applying direct pressure [45], particularly when HFS or more realistic models are being used, with no major financial expenditure [46]. The study by Orlas et al. [45] was conducted through a master class followed by a practical component, employing Z-Medica's training kits, which include certified manikins. Additionally, the study by Villegas et al. [46] also employed manikins as simulators.

Regarding the limitations of the study, it should be noted that the intervention was a pre-post design with expected improvements after the training and a late assessment at three months. Nonetheless, this is the first study to incorporate a low-cost simulator with regular nursing faculty materials in an HFS scenario, which demonstrated reliability and validity in evaluating advancement in knowledge level, self-efficacy, and haemorrhage control. Although our results demonstrate reliability and validity, it is important to note that the study design has several limitations and is not adequate for establishing causality. Therefore, in future studies, it would be desirable to perform a randomised trial that measures the retention of this training for a longer period than three months and expand the sample to other populations such as medical students, other healthcare professionals, civilian populations, or laypeople. Finally, another limitation of this study to consider was the use of tourniquets solely as haemorrhage control methods; hence, it might be worthwhile paying attention to other approaches such as pressure, wound packing, and others.

## 5. Implications for Nursing Management

In general, it seems that educating and training healthcare professionals and students is effective in promoting self-efficacy, knowledge retention, and ensuring patient survival while controlling and managing haemorrhages [47–51]. However, both laypeople and healthcare professionals continue to lack the necessary knowledge and expertise to utilise tourniquets properly [52], and thus, our findings may help healthcare educators and supervisors to find a suitable approach to foster training among their professionals and students in effective bleeding control. As previously stated in the literature, it may be worth noting that this basic training could serve as the initial step in a train-the-trainer cascade, allowing for more knowledge distribution and reaching a broader audience [20, 53].

## 6. Conclusion

The effectiveness of a low-cost task simulator within an HFS scenario for haemorrhage control training in nursing students was evaluated in this study, which revealed a significant improvement in the practical skills and knowledge retention of the students who used the simulator. This was evidenced by a reduction in the time required to apply a tourniquet and the millilitres of blood lost in the simulator, whereas knowledge and perceived self-efficacy were increased. Although a decrease in the performance of the students was observed after three months, they still maintained higher results than those obtained before the training and close to those obtained immediately after the high-fidelity clinical simulator training. Overall, these findings indicate that using a low-cost task simulator within an HFS can be a valuable resource for training nursing students in haemorrhage control, with effects that are comparable to additional simulators and persist over time, though reinforcement of its application may be required in the future.

## Figures and Tables

**Table 1:** Simulation training session of bleeding control.

PRE	Prebriefing	Simulation		Debriefing	POST	
		HFS components	Simulated scenario		IPA	3M
Knowledge questionnaire (SBEAT)	Military nurses provide 90-minute specialised training based on the first-care provider model	Modality (exsanguinating haemorrhage simulator) Fidelity (realism through simulation equipment, setting, and scenario)	Haemorrhage-control task simulator integrated into a traumatic multiple-incident scenario (including an exsanguinating haemorrhage)	Plus-delta approach using double-barrelled questioning (problem-solving and critical thinking through informed learner self-assessment, and managing perception mismatches)	Knowledge questionnaire (SBEAT) Self-efficacy questionnaire (5-point Likert scale)	Knowledge questionnaire (SBEAT)
Self-efficacy questionnaire (5-point Likert scale)			Standardised emergency equipment (including tourniquet)			Self-efficacy questionnaire (5-point Likert scale)
Practical skills (time, exsanguinated volume)			Practical skills (time, exsanguinated volume)			Practical skills (time, exsanguinated volume)

3M: 3-month assessment; HFS: high-fidelity simulation; IPA: postassessment; PRE: preassessment; POST: postassessment; SBEAT: Bleed Education Assessment Tool.

**Table 2:** Baseline demographic characteristics of participants.

Variable	N/M	%/SD
Gender		
Male	22	21.36
Female	81	78.64
Age (years)	23.34	5.83
Knowledge (0–100)	70.58	10.24
Self-efficacy (0–100)	45.83	21.69
Time (s)	76.38	41.28
Volume (mL)	573.30	246.10

**Table 3:** Comparison of the variables knowledge, self-efficacy, time, and volume pre- and postintervention.

Variable	PRE (M (SD))	POST (M (SD))	3M (M (SD))	PRE-POST sig. (*Z*)	PRE-3M sig. (*Z*)	POST-3M sig. (*Z*)
Knowledge (%)	70.58 (10.24)	79.51 (8.53)	76.70 (8.95)	0.000 (−5.84)	0.000 (−4.82)	0.011 (−2.55)
Self-efficacy (%)	45.83 (21.69)	77.37 (15.36)	68.50 (15.19)	0.000 (−8.53)	0.000 (−8.23)	0.000 (−5.92)
Time (s)	76.38 (41.28)	41.69 (27.13)	47.12 (28.88)	0.000 (−6.65)	0.000 (−5.00)	0.013 (−2.48)
Volume (mL)	573.30 (246.10)	325.15 (179.83)	387.38 (199.10)	0.000 (−6.98)	0.000 (−5.23)	0.005 (−2.84)

3M: 3-month assessment; PRE: preassessment; POST: postassessment.

## Data Availability

The data that support the findings of this study are available on request from the corresponding author. The data are not publicly available due to privacy or ethical restrictions.
